# LINC00893 inhibits the progression of prostate cancer through miR-3173-5p/SOCS3/JAK2/STAT3 pathway

**DOI:** 10.1186/s12935-022-02637-4

**Published:** 2022-07-10

**Authors:** Chuigong Yu, Yu Fan, Yu Zhang, Lupeng Liu, Gang Guo

**Affiliations:** Department of Urology, The Third Medical Center, Chinese People’s Liberation Army General Hospital, No. 69, Yongding Road, Haidian District, Beijing, 100039 China

**Keywords:** LINC00893, Prostate cancer, miR-3173-5p, SOCS3, JAK2/STAT3 signaling pathway

## Abstract

**Background:**

Prostate cancer (PCa) is one of the most common malignant tumors in the male urinary system. In recent years, the morbidity and mortality of PCa have been increasing due to the limited effects of existing treatment strategies. Long non-coding RNA (lncRNA) LINC00893 was reported to inhibit the proliferation and metastasis of papillary thyroid cancer cells, but its role in PCa has not been reported. This study aims to investigate the role and underlying mechanism of LINC00893 in regulating the progression of PCa cells.

**Methods:**

We first compared LINC00893 expression levels between PCa tissues and normal prostate tissues through TCGA database. The relative LINC00893 expression levels were further validated in 66 pairs of PCa tissues and para-cancerous normal tissues, as well as in PCa cell lines. Gain-of-function experiment was performed by transfecting PCa cell with LINC00893 expression vector, and CCK (Cell count kit)-8, 5-Ethynyl-2′-deoxyuridine (EdU) incorporation, colony information and transwell assays were conducted to assess the functional phenotypes. Dual-luciferase reporter, RNA-binding protein immunoprecipitation (RIP) and RNA pull-down assays were performed to evaluate the molecular interactions.

**Results:**

LINC00893 was downregulated in PCa tissues and cell lines, and patients with low expression of LINC00893 were associated with a poorer overall survival rate. LINC00893 overexpression hindered the proliferation, epithelial-mesenchymal transition (EMT) as well as the migratory ability of PCa cells, and suppressed the tumorigenesis of PCa cells in nude mice. We further demonstrated that LINC00893 acted as a sponge for miR-3173-5p and inhibited its activity, which in turn regulated the suppressor of cytokine signaling 3 (SOCS3)/Janus Kinase 2 (JAK2)/signal transducer and activator of transcription 3 (STAT3) signaling axis.

**Conclusions:**

Our study demonstrated that LINC00893 suppresses the progression of PCa cells through targeting miR-3173-5p/SOCS3/JAK2/STAT3 axis. Our data uncovers a novel tumor-suppressor role of LINC00893 in PCa, which may serve as a potential strategy for targeted therapy in PCa.

**Grapical Abstract:**

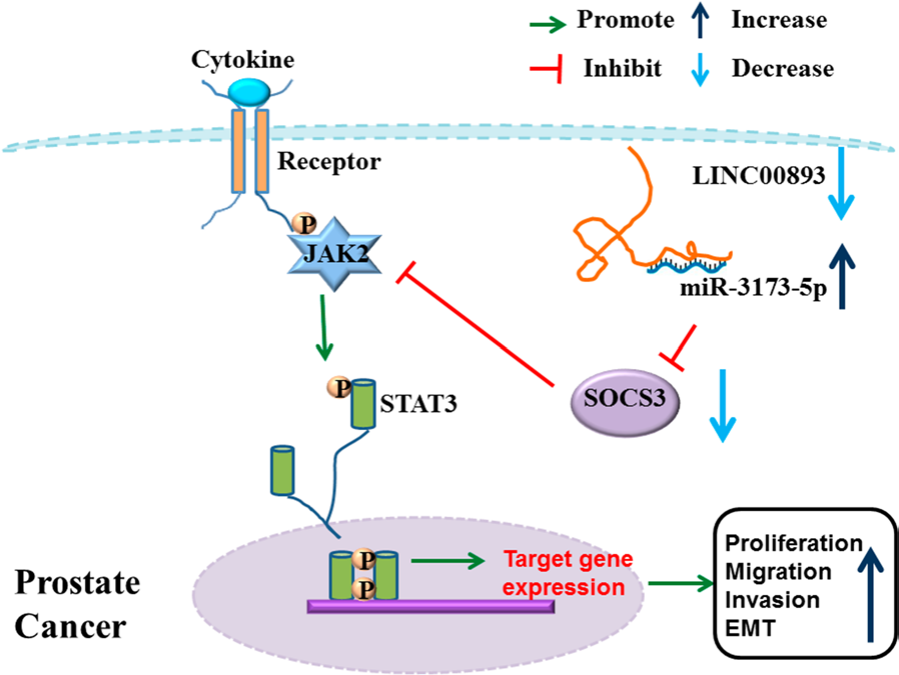

**Supplementary Information:**

The online version contains supplementary material available at 10.1186/s12935-022-02637-4.

## Introduction

Prostate cancer (PCa) is the most common epithelial malignant tumor in the male urinary system [1, 2]. Advanced age, heredity, obesity and environmental pollution are the crucial risk factors for PCa [3–6]. Surgical resection, androgen deprivation therapy, radiation therapy and chemotherapy remain as the mainstays for PCa treatment [7]. However, tumor recurrence and drug resistance development limit the success of the current strategies. Therefore, exploring molecular mechanisms underlying the pathogenesis and progression of PCa could identify the potential molecular targets for the formulation of novel therapies.

Non-coding RNAs (ncRNAs) have been identified in most organisms, and are implicated in regulating the malignant phenotypes of cancer cells [8, 9]. NcRNAs can be classified into long ncRNAs (lncRNAs) (> 200 nt) and short ncRNAs (sncRNA) (< 30 nt) according to the transcript length. SncRNA contains microRNAs (miRNAs) and short interfering RNAs (siRNAs) [8–13]. LncRNAs and microRNAs are implicated in the initiation and progression of various cancers including prostate cancer [14], glioma [15] and breast cancer [16], and their deregulations have been proposed as potential biomarkers and therapeutic targets [17, 18]. Accumulating evidence reveals that lncRNAs usually function as competing endogenous RNAs (ceRNAs) to inhibit the activities of miRNAs [19]. For example, LncRNA NEAT1 serves as sponges for both miR-34a-5p and miR-204-5p, which promotes the docetaxel resistance in PCa cells [20]. LncRNA CASC15 targets miR-200a-3p and enhances the metastatic ability of PCa cells [21]. LncRNA AATBC accelerates the PCa progression by regulating miR-1245b-5p/calmodulin-dependent serine protein kinase (CASK) axis [22]. However, the potential role and regulatory mechanism of LINC00893 in PCa progression remain to be elucidated.

Non-receptor tyrosine kinase Janus kinase 2 (JAK2) and transducer and activator of transcription 3 (STAT3) are signal mediators stimulated by cytokines [23, 24]. Upon cytokine engagement, JAK2 is phosphorylated by the cytokine receptor and phosphorylated JAK2 activates transcription factor STAT3 and promotes the nuclear translocation, which regulates a wide spectrum of target genes involved in proliferation, differentiation and epithelial-mesenchymal transition (EMT) [25–27]. The activity of JAK2/STAT3 signaling pathway is inhibited by the suppressor of cytokine signaling (SOCS) family. The SOCS family contains seven SOCS members as well as cytokine-inducible SH2-containing protein (CIS) [28, 29]. Among the SOCS members, SOCS3 has a higher affinity for JAK2, and it can induce the proteasome-dependent degradation of JAK2 and hinder the binding of STAT3 to gene promoters by modifying its conformation [30, 31]. Therefore, SOCS3 serves as the most critical negative regulator of JAK2/STAT3 signaling pathway [32–34]. Recent studies have shown that SOCS3/JAK2/STAT3 signaling axis is implicated in the progression of multiple cancers. For instance, SOCS3/JAK2/STAT3 signaling axis mediates the inhibitory role of piperlongumine in the progression of osteosarcoma [35]. SOCS3/JAK2/STAT3 signaling pathway also regulates the malignant progression of hepatocellular carcinoma [36]. Furthermore, the dysregulation of SOCS3/JAK2/STAT3 signal axis is associated with epithelial-mesenchymal transition (EMT) and metastatic ability of gallbladder cancer cells [37]. However, the regulatory roles of SOCS3/JAK2/STAT3 signaling axis in PCa remain to be investigated.

In the current study, we found that LINC00893 was downregulated in PCa tissues and cell lines, and patients with low expression of LINC00893 were associated with a poorer overall survival rate. Gain-of-function experiments showed that LINC00893 overexpression suppressed the proliferation, EMT as well as the migratory ability of PCa cells, and hindered the tumorigenesis of PCa cells in nude mice. We further demonstrated that LINC00893 acted as a sponge for miR-3173-5p and inhibited its activity, which in turn regulated SOCS3/JAK2/STAT3 signaling axis to modulate the malignant phenotypes in PCa cells. Our study uncovered a tumor-suppressor role of LINC00893 in the malignant progression of PCa cells.

## Materials and methods

### Tissue samples

A total of 66 pairs of PCa tissues and matched para-cancerous tissues (stage B2 tumors: 8, stage C tumors: 35 and stage D tumors: 23) were obtained from Department of Urology, The Third Medical Center of Chinese People's Liberation Army General Hospital from February 2018 to September 2020. These patients (Gender: 66 males, Age: 65 to 82 years old) were diagnosed with PCa by pathological examination. None of the patients had undergone radiotherapy and chemotherapy before PCa surgical resection, and these patients had complete medical records since admission. Patients with other chronic diseases including hypertension, diabetes, metabolic diseases, immune diseases and other tumors were excluded from the study. All the patients signed informed consent. This study was approved by the Medical Ethics Committee of The Third Medical Center of Chinese People's Liberation Army General Hospital.

### Cell culture and transfection

All PCa cells (PC-3, DU145, VCaP and LNCaP) as well as human normal prostate epithelial cells (RWPE-1) were obtained from American Type Culture Collection (ATCC, Rockville, MD, USA). PC-3, DU145 and RWPE-1 cells were grown in F-12K basic medium (Invitrogen, California, USA), Eagle’s Minimum Essential medium (EMEM, Gibco, California, USA) and Keratinocyte serum free medium (Invitrogen), respectively. VCaP and LNCaP cells were cultured in RPMI 1640 basic medium (Gibco). All culture media were complemeted with 10% fetal bovine serum (FBS, Gibco) as well as 100 U/mL penicillin and 100 µg/mL streptomycin (Gibco) except for RWPE-1. The cells were maintained in a humidified incubator under conditions of 37 °C with 5% CO_2_.

The LINC00893 sequence was cloned into pcDNA3.1 vector for LINC00893 overexpression (pcDNA3.1-LINC00893). pcDNA3.1-LINC00893 plasmid and pcDNA3.1 empty vector were purchased from Genechem Co., Ltd (Shanghai, China). MiR-3173-5p mimic, miR-3173-5p inhibtor, siRNA targeting SOCS3 (si-SOCS3) and the corresponding negative controls were purchased from RiboBio Co., Ltd. (Guangzhou, China). Cell transfection was performed using Lipofectamine® 3000 reagent (Thermo Fisher Scientific, L3000001). In 6 well plate, 60% confluent cells were transfected with 200 nM of microRNA mimic/inhibitor or 6 μg of plasmid according to the manufacturer’s instructions. Transfected cells were subjected to subsequent analysis 48 h post-transfection.

The sequences of the transfected molecules are as follows: miR-3173-5p-mimic: 5ʹ-UGCCCUGCCUGUUUUCUCCUUU-3ʹ; miR-3173-5p-inhibtor: 5ʹ-AAAGGAGAAAACAGGCAGGGCA-3ʹ.

### LINC00893 stable expression cell line generation

To generate cell line with stable LINC00893 overexpresison, PC-3 cells were infected with recombinant lentivirus carrying sequence LINC00893 (PC-3-LINC00893) or matched control (PC-3-vector) (Shanghai Genechem Co., Ltd., China). 48 h post infection, cells stably overexpressing LINC00893 were selected using 1.0 μg/mL puromycin for 2 weeks. The stable cells lines were maintained in 500 ng/mL puromycin.

### Real-time quantitative PCR analysis (RT-qPCR)

Total RNA samples from tissues and cells were extracted using Trizol method as previously described [38]. For tissue samples, 100 mg tissue blocks were fully ground in liquid nitrogen before the addition of Trizol reagent. RNA concentration was measured using NanoDrop, and 5 μg total RNA was used for reverse transcription by cDNA Reverse Transcription Kit (Invitrogen, K1691). SYBR premix EX TAQ II kit (Takara, Dalian, China) was used for the quantitative analyses of cDNA samples on 7500 Real Time PCR System (Applied Biosystems, Carlsbad, CA, USA). The relative expression levels were calculated according to 2^–∆∆Ct^ method by normalizing to Glyceraldehyde-3-phosphate dehydrogenase (GAPDH). Three independent assays were performed for each sample. qPCR primers were synthesized by Sangon Biotechnology Co., Ltd. (Shanghai, China): LINC00893 (Forward: 5ʹ-AGTCGGCTCACCAATTCGT-3ʹ, Reverse: 5ʹ-CAGGCACCCTATCCCGAAG-3ʹ); U6 (Forward: 5ʹ-CTCGCTTCGGCAGCACA-3ʹ, Reverse: 5ʹ-AACGCTTCACGAATTTGCGT-3ʹ); GAPDH (Forward: 5′-GACAGTCAGCCGCATCTTCT-3′, Reverse: 5′-GCGCCCAATACGACCAAATC-3′); miR-3173-5p (Forward: 5ʹ-TGCCCTGCCTGTTTTCTCC-3ʹ, Reverse: 5′-GAACATGTCTGCGTATCTC-3ʹ); SOCS3 (Forward: 5ʹ-CCTGCGCCTCAA GACCTTC-3ʹ, Reverse: 5ʹGTCACTGCGCTCCAGTAGA A-3ʹ).

### CCK-8 cell proliferation assay

PCa cells with different treatment were seeded in 96-well-plates (2000 cell/well). Cells were cultured under conditions of 37 °C with 5% CO2 for 0 h, 24 h, 48 h, 72 h and 96 h. Subsequently, 10 μL CCK-8 reaction reagent (Solarbio, CA1210, Beijing, China) was added into each well at indicated time point and the cells were incubated for 4 h. Finally, the absorbance at 450 nm in each condition was recorded using a microplate reader (Bio-Rad, Hercules, USA) [39]. CCK-8 cell proliferation assay was conducted with three independent biological replicates for each condition.

### 5-Ethynyl-2-deoxyuridine (EdU) incorporation assay

EdU incorporation was conducted using the EdU assay kit (C10310-1, RiboBio, Guangzhou, China). Briefly, PC-3 and LNCaP cells were seeded into 24-well-plates (1 × 10^5^ cells/well) and cultured overnight with F-12K and RPMI 1640 medium, respectively. The cells were transfected with pcDNA3.1-LINC00893 overexpression plasmids or pcDNA3.1-vector for 48 h. After transfection, cells were incubated with 200 μL 50 μm EdU for 2 h, followed by fixation with 2% paraformaldehyde (PFA) for 20 min. 2 mg/mL glycine was used to neutralize PFA. After that, cell membrane was permeabilized by adding 100 μL PBS containing 0.1% Triton X-100 for 10 min. After the removal of permeabilization solution, cells were stained with 100 μL Apollo staining solution for 30 min, followed with the nuclear staining by 4′,6-diamidino-2-phenylindole (DAPI) solution for 20 min at ambient temperature in the dark. The fluorescent images were captured under a fluorescence microscope (Nikon, Japan) 48 h after mounting [40]. Three independent replicates were imaged and analyzed in the EdU assay.

### Colony formation assay

PC-3 and LNCaP cells with different treatment were resuspended in fresh medium and then seeded into the 6-well-plates (1000 cells/well). The cells were grown under the condition of 37 °C and 5% CO_2_ for 14 days, and the medium was replaced every three days. Cells were fixed by 4% paraformaldehyde for 20 min, and stained using Giemsa Stain Kit (Abcam, ab150670) for 20 min at room temperature. Colony morphology were photographed and colony number was counted under Leica AM6000 microscope [41]. The colony formation assay was conducted with three independent biological replicates.

### Cell migration and invasion assay

Transwell assays were employed to assess cell migration and invasion abilities according to the method in a previous study [42]. PC-3 and LNCaP cells in 6-well-plates were transfected with pcDNA3.1-LINC00893, with or without miR-3173-5p inhibitor/mimic or SOCS3 siRNA. 48 h post transfection, cells were collected and resuspended in serum-free culture medium. For invasion assay, the 24-well transwell upper chamber (Sigma, Germany) was pre-coated with 500 μg/ml Matrigel (BD Biosciences, Bedford, MA). For migration assay, the upper chamber was not coated with Matrigel. A total of 5 × 10^4^ cells were seeded into the upper chamber in serum-free medium, and 500 μL culture medium with 10% FBS was added in the lower chamber. After 48 h, cells in the upper chamber were wiped off by swabs, and cells on the membranes were fixed with 4% paraformaldehyde for 10 min and stained with 0.5% crystal violet for 20 min. The migrating and invading cells were counted under a Leica DC 300F microscope. Cell migration and invasion assays were carried out in three independent biological replicates. For each replicate, six visual fields were randomly selected for cell counting, and the mean cell number of six fields was calculated as the relative migrating cell number.

### Western blot (WB)

Western blot analysis of protein abundance was performed as previously described [42]. Cells were lysed in ice-cold RIPA Lysis Buffer for 15 min. Cell lysates were centrifuged at 12,000 rpm for 10 min and the supernatants were collected. Protein concentration in the supernatant was determined by a BCA protein assay kit (Thermo Fisher Scientific, Waltham, MA, USA). An equal volume of 2X SDS loading buffer was added into cell lysates and the protein samples were heated at 100 °C for 10 min. 40 μg of protein sample was separated in SDS-PAGE system and further transferred to polyvinylidene fluoride (PVDF) membrane (BioRed, USA). Subsequently, the PVDF membrane was blocked in 5% skimmed milk for 1 h at room temperature and incubated with primary antibodies at 4 °C overnight: anti-E-Cadherin (CST, #3195, 1:1000), anti-N-Cadherin (CST, #14,215, 1:1000), anti-vimentin (CST, #5741, 1:1000), anti-GAPDH (CST, #2118, 1:2000), anti-SOCS3 (CST, #52,113, 1:1000), anti-Phospho-JAK2 (CST, #3771, 1:1000), anti-JAK2 (CST, #74,987, 1:1000) anti-p-STAT3 (CST, #9145, 1:1000) and anti-STAT3 (CST, #12,640, 1:1000). The membrane was washed 3 times with 1 × TBST buffer (5 min each time), and further incubated with HRP-labeled secondary antibody (Anti-rabbit IgG, HRP-linked Antibody: CST, #7074, 1:2000; Anti-mouse IgG, HRP-linked Antibody: CST, #7076, 1:2000) for 1 h at room temperature. After washing, the protein bands were developed using enhanced chemiluminescence reagent (ECL, Thermo Fisher Scientific) and the relative protein abundance was quantified employing Image J software. The Western blot was conducted using three independent biological samples, and the quantification data were summarized in Additional file 1: Table S1.

### RNA subcellular isolation

Cell fractioning was performed using RNA Subcellular Isolation Kit (25,501, Active Motif, California, USA.). Briefly, cells growing in 150 mm plates at 90% confluence were harvested by trypsin and centrifuged at 14,000 rpm for 5 min. Cell pellets were resuspended in 120 μL complete lysis buffer and incubated for 10 min on ice. The cell lysates were centrifuged at 14,000 rpm for 5 min. The supernatant containing cytoplasmic fraction and the pellet containing the nuclear faction were used for RNA extraction with Trizol reagent. The resulted RNA was dissolved in DEPC water, and the reverse-transcription and qRT-PCR analysis of U6 small nuclear RNA (U6 snRNA), GAPDH and LINC00893 were performed based on the procedures described in RT-qPCR section. U6 snRNA and GAPDH were used as the markers of the nucleus and cytoplasm, respectively. Three independent biological replicates were collected in RNA Subcellular Isolation assay.

### Dual-luciferase reporter assay

Dual-luciferase reporter assay was conducted according to the method in a previous study [43]. The binding sites between LINC00893 and miR-3173-5p, or miR-3173-5p and SOCS3 mRNA 3’ UTR (Untranslated region) were predicted through Starbase database. The sequences of LINC00893 containing the wide-type (WT) or mutant (MUT) binding sites were cloned into the pmirGLO luciferase reporter vector (Promega, Madison, WI, USA) to construct WT (pmirGLO-WT-LINC00893) or MUT (pmirGLO-MUT-LINC00893) luciferase reporter for LINC00893. Similar procedures were used to generate WT (pmirGLO-WT-SOCS3) and MUT (pmirGLO-MUT- SOCS3) luciferase reporter of SOCS3. The WT or MUT luciferase reporter and Renilla luciferase (Rluc) control plasmid were co-transfected into PC-3 and LNCaP cells in the presence of miR-3173-5p mimic or miR-NC. 48 h after transfection, the relative luciferase activity was assessed using the Dual-Luciferase® Reporter Assay System (Promega, E1910) on a luminescence microplate reader (Infinite 200 PRO; Tecan). Renilla luciferase signal was used as internal reference. Dual-luciferase reporter assay was conducted with three independent samples.

### RNA-binding protein immunoprecipitation (RIP) assay

RIP assay was conducted according to the method reported in a previous study [44]. The functional interaction between LINC00893 and miR-3173-5p was assessed employing Magna RIP™ RNA-Binding Protein Immunoprecipitation Kit ( Millipore, Billerica, MA, USA) according to the manufacturer's instructions. Briefly, PC-3 and LNCaP cells were lysed with RIP lysis buffer with RNase inhibitor (Takara) as well as protease inhibitor cocktail (Thermo Fisher Scientific). The cell lysate was centrifugated at 12,000 rpm for 30 min to remove cell debris. Subsequently, 100 μL magnetic beads conjugated with IgG (Millipore) or anti-Argonaute 2 (Ago2) antibodies (Millipore) were added to 1000 μL cell lysate, and 10% cell lysate was reserved as the input. The mixture was incubated overnight at 4 °C, followed by 4 times washing with washing buffer. RNA samples in the input or precipitated by the beads were extracted and quantified as described in RT-qPCR section. Three independent biological replicates were carried out for RIP assay.

### RNA pull-down assay

Cells in 6-well plates were transfected with 50 nM biotin-labeled miR-3173-5p or miR-NC for 48 h. About 5 million cells were lysed using 1000 μL cell lysis buffer (Ambion, Austin, TX, USA), followed by the centrifugation at 14,000 rpm for 10 min. 100 μL supernatant was take out as the input, and the remaining supernatant was incubated with 100 μL M-280 streptavidin magnetic beads (Sigma) pre-blocked by yeast tRNA and RNase-free BSA (Sigma) at 4 °C overnight. The magnetic beads were rinsed 4 times with lysis buffer, and RNA samples was extracted and analyzed by RT-qPCR [45]. Three independent assays were conducted for RNA pull-down assay.

### Xenograft tumorigenesis assay

A total of 1 × 10^6^ PC-3 cells stably expressing LINC00893 (PC-3-LINC00893) or the matched control cells (PC-3-vector) were subcutaneously inoculated into the left flank of 6-week-old male NOD/SCID mice (n = 6 in each group, Gempharmatech. Co., Ltd,). The tumor volume was examined every 5 days. On the 30 th day, tumor-bearing mice were sacrificed using pentobarbital solution (150 mg/kg, i.p.), followed by cervical dislocation. The subcutaneous tumors were removed and weighed. The tumor tissues were fixed and used for immunohistochemistry (IHC) staining, or RNA and protein sample analysis. The animal study was approved by the The Laboratory Animal Ethics Committee of The Third Medical Center of Chinese People's Liberation Army General Hospital.

### Immunohistochemistry (IHC)

Xenograft tumor tissues were fixed with 4% paraformaldehyde and then embedded in paraffin. The paraffin-embedded tissues were cut into 5 μm sections and fixed on glass slides. The tissue sections were baked at 65 °C for 2 h and then submerged in xylene for deparaffinization, which was followed by dehydration with alcohol gradient (100%–100%–95%–90%–80%–70%). Subsequently, pH 6.0 citrate buffer was added to the tissue sections for antigen retrieval. After that, 1% H_2_O_2_ was added to tissue sections for 20-min incubation. After washing with 1xTBST buffer, the sections were incubated with primary antibodies: anti-Ki67 (CST, #9027, 1: 200), anti-SOCS3 (Santa, # sc-518020, 1:200) overnight at 4 °C. After washing with TBST buffer for 3 times, the sections were further incubated with secondary antibody (GE Healthcare, Piscataway, NJ, USA) for 1 h at room temperature. The color was developed using DAB kit (Maxin, Fuzhou, China) for 5 min, which was followed by the counterstaining using hematoxylin for 1 min. The tissue sections were dehydrated and sealed with neutral resin, and the stained samples were imaged under Leica DC 300F microscope.

### Statistical analysis

Statistical analyses were conducted using SPSS 13 software and Graphpad Prism 6.0 software was employed to generate the figures. Data was presented in the form of mean ± standard deviation (SD). The difference between the experimental group and control group was analyzed by student’s t-test. The comparison of differences among multiple groups was performed using one-way analysis of variance (ANOVA), with Tukey’s post hoc test for pairwise comparison. The difference in survival time of patients in LINC00893-low and LINC00893-high groups was compared by log-rank test. Two-way ANOVA was employed to compare the differences in CCK-8 cell proliferation assay and tumor growth volume in tumorigenesis assay at different time points. Spearman correlation analysis was performed to assess correlation between the expression levels of two molecules. Generally, *P* < 0.05 was considered to be statistically significant.

## Results

### LINC00893 is downregulated in PCa tissues and cell lines

**Fig. 1:**
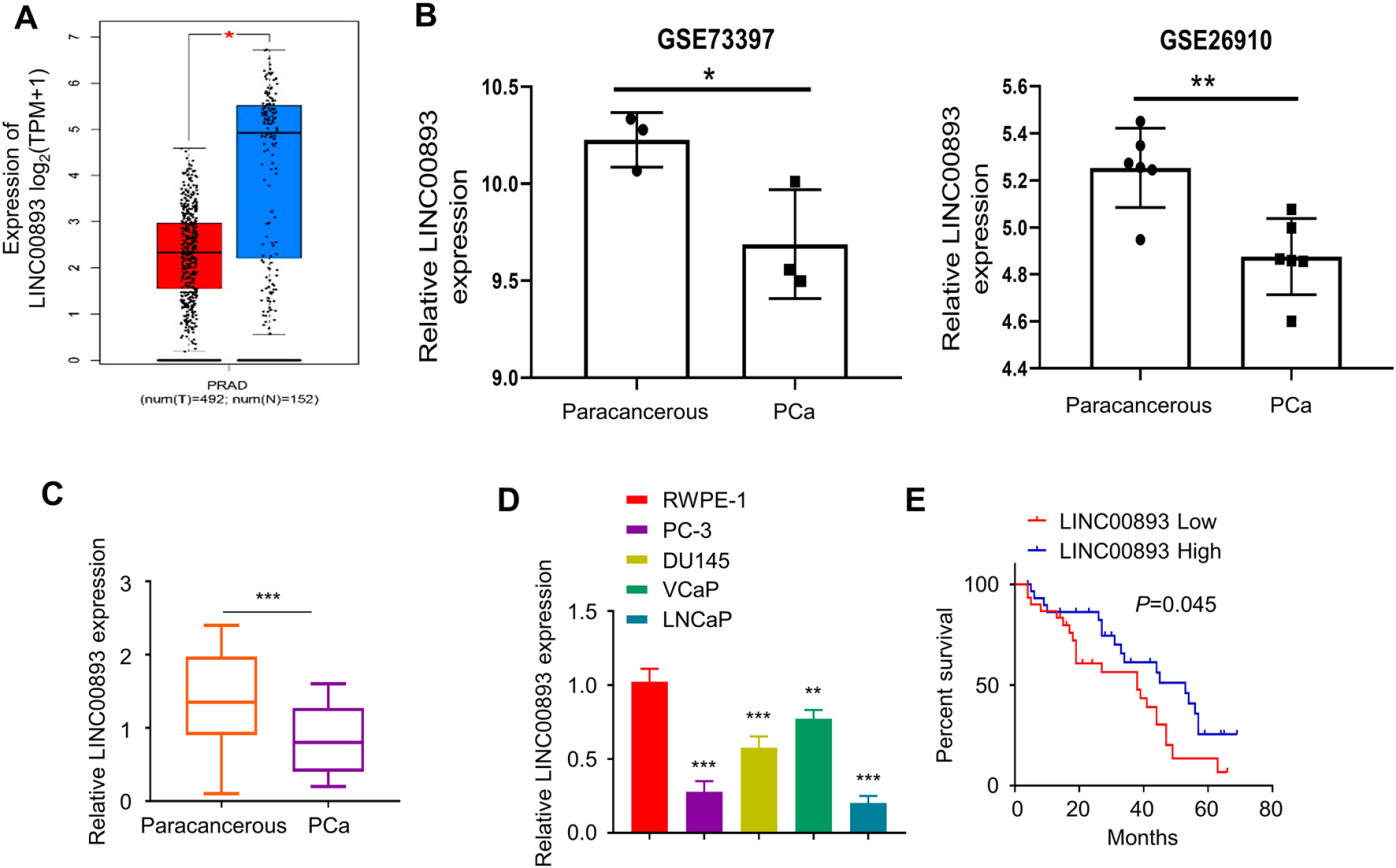
LINC00893 is downregulated in PCa tissues and cell lines. **A**,** B** LINC00893 expression levels in PCa tissues and paired para-cancerous tissues were compared in TCGA database (**A**), and in GSE73397 & GSE26910 datasets from GEO database (**B**). **C** The relative LINC00893 expression level was examined in 66 pairs of PCa tissues and para-cancerous tissues by RT-qPCR. **D** LINC00893 expression levels were examined in PCa cell lines (PC-3, DU145, VCaP and LNCaP) and human normal prostate epithelial cell line (RWPE-1) by RT-qPCR. **E** 66 PCa patients were divided into LINC00893_low_ (n = 33) and LINC00893_high_ (n = 33) group according to the median value of LINC00893 expression. Kaplan–Meier survival analysis was used to evaluate the overall survival rate of patients in the two groups. Three independent assays were performed with three technical replicates in **C** and **D**. The error bars are defined as s.d. *, P < 0.05, **, P < 0.01, and ***, P < 0.001

To investigate the expression pattern of LINC00893 in PCa, we first compared LINC00893 expression levels between PCa tissues and normal prostate tissues using TCGA (The Cancer Genome Atlas) database, and the GSE73397 and GSE26910 datasets from GEO (Gene expression omnibus) database. The analysis showed that LINC00893 was significantly downregulated in PCa tissues when compared with the para-cancerous tissues (Fig. 1A, B). To validate this finding, we collected 66 pairs of PCa tissues and adjacent normal tissues, and examined LINC00893 expression by RT-qPCR. Consistently, the results showed that LINC00893 expression level was significantly reduced in PCa tissues (Fig. 1C). Furthermore, the relative LINC00893 expression level in PCa cell lines (PC-3, DU145, VCaP and LNCaP) was also lower than that of human normal prostate epithelial cell line (RWPE-1) (Fig. 1D). To explore the ralationship between LINC00893 expression level and the survival of PCa patients, 66 PCa patients were divided into LINC00893 low-expression group (n = 33) and LINC00893 high-expression group (n = 33) based on the median value of LINC00893 expression. Kaplan–Meier survival analysis showed that patients in LINC00893 low-expression group were associated with a significantly poorer overall survial (Fig. 1E). In addition, the analyses of the relationship between LINC00893 expression and clinicopathological parameters demonstrated that a high LINC00893 expression level was closely associated with TNM staging and distant metastasis, but showed no correlation with age, gender, tumor size and tumor differentiation degree (Table 1). Collectively, the above results indicate that LINC00893 functions as a tumor-suppressor in PCa.

**Table 1:** The correlation between the expression of LINC00893 and the clinicopathological features of PCa

Variable	LINC00893		P value
	Low (n = 33)	High (n = 33)	
Age (years)			0.614
≤ 60	19	21	
> 60	14	12	
Tumor size			0.083
≤ 5	22	15	
> 5	11	18	
TNM stage			0.026
I/II	19	10	
III/IV	14	23	
Distant metastasis			0.048
Yes	11	19	
No	22	14	
Tumor differentiation			0.138
High/middle	12	18	
Low	21	15	

### LINC00893 overexpression inhibits the proliferation, migration, invasion and EMT of PCa cells

**Fig. 2:**
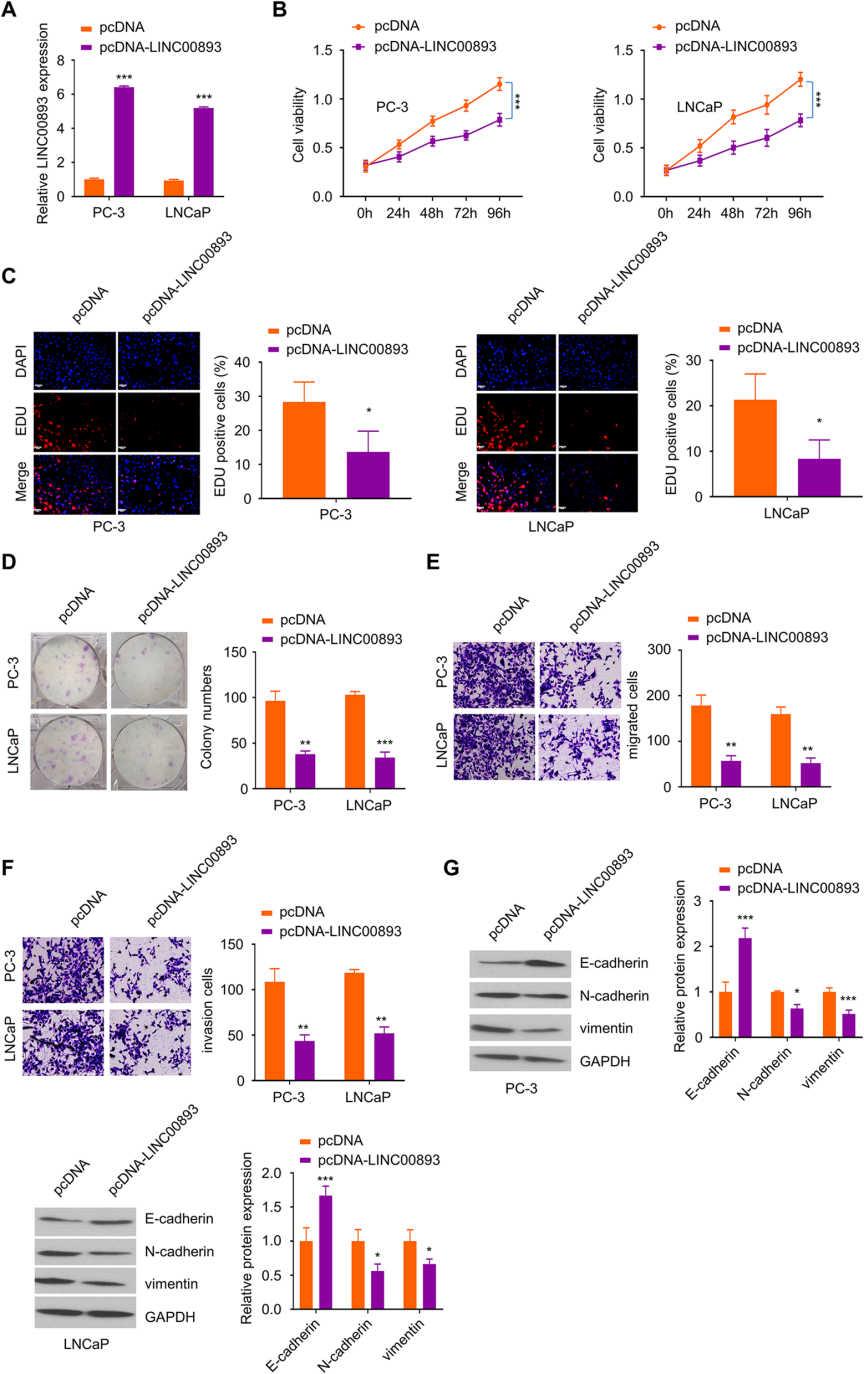
LINC00893 functions as a tumor suppressor factor to inhibit the proliferation, migration, invasion and EMT of PCa cells. PC-3 and LNCaP cells were transfected with pcDNA3.1-LINC00893 plasmids or pcDNA3.1-vector for 48 h. **A** LINC00893 expression level was examined by RT- qPCR in cells transfected with pcDNA3.1-LINC00893 or vector. **B** CCK-8 proliferation assay in cells transfected with pcDNA3.1-LINC00893 or vector was perfromed at indicated time points. **C** EdU incorporation assay in cells transfected with pcDNA3.1-LINC00893 or vector. Scale bar: 50 μM. **D** Colony formation assay in cells transfected with pcDNA3.1-LINC00893 or vector. **E**,** F** Transwell migration assay (**E**) and invasion assay (**F**) in cells transfected with pcDNA3.1-LINC00893 or vector. **G** The protein levels of E-cadherin and mesenchymal markers N-cadherin and vimentin were assessed by Western blot in cells transfected with pcDNA3.1-LINC00893 or vector. Three independent assays were performed with three technical replicates. The error bars are defined as s.d. *, P < 0.05, **, P < 0.01, and ***, P < 0.001

To investigate the functional role of LINC00893 in PCa cells, we conducted gain-of-function experiments by transfecting PCa cells with LINC00893 expression vector. PC-3 and LNCaP cell lines with the lowest LINC00893 expression were selected for overexpression experiments. RT-qPCR analysis showed that the transfection of pcDNA3.1-LINC00893 plasmid could effectively upregulate LINC00893 level in both cell lines (Fig. 2A). Subsequently, CCK-8 cell proliferation experiment and EdU incorporation assay demonstrated that LINC00893 overexpression inhibited the cell proliferation ability and reduced the percentage of cells actively replicating the DNA (Fig. 2B and C). Consistently, colony formation assay showed the impaired clonogenic ability of PCa cells upon LINC00893 overexpression (Fig. 2D). To further examine the migratory phenotype, we performed Transwell migration and invasion assays in cells transfected with LINC00893 expression vector or empty vector. LINC00893 overexpression suppressed the migration and invasion ability in both PC-3 and LNCaP cell lines (Fig. 2E and F). As cell migration is closely associated with EMT, we next examined the effect of LINC00893 on the expression of EMT markers. LINC00893 overexpression significantly downregulated mesenchymal cell markers N-cadherin and vimentin, while the epithelial marker E-cadherin was upregulated (Fig. 2G). Together, these data suggest that a high level of LINC00893 expression impairs the malignant phenotype of PCa cells.

### LINC00893 sponges miR-3173-5p and suppresses its expression

**Fig. 3:**
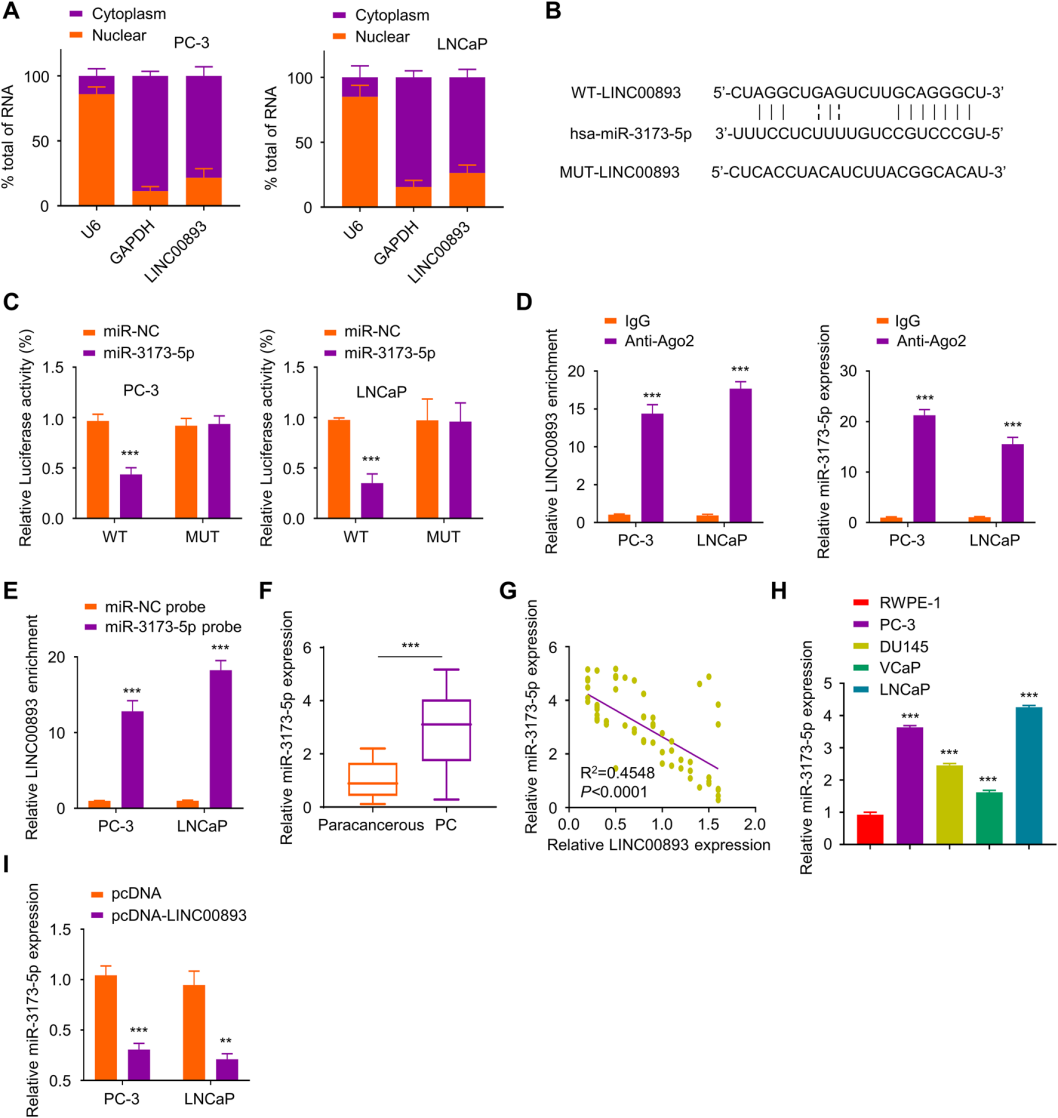
LINC00893 binds to miR-3173-5p and inhibits its expression. **A** Relative LINC00893 levels were quantified by RT-qPCR in the cytoplasmic and nuclear fractions of PC-3 and LNCaP cells. U6 and GAPDH were used as marker for the nuclear and cytoplasmic fraction respectively. **B** The binding sites between LINC00893 and miR-3173-5p were predicted from Starbase database. **C** Dual luciferase reporter assay was performed using pmirGLO-WT-LINC00893 and pmirGLO-MUT-LINC00893 reporter in the presence of miR-3173-5p mimic or miR-NC. Renilla luciferase control plasmids were co-transfected into cells, and the relative firefly luciferase activity in the reporter was normalized to renilla luciferase activity in the control plasmid. **D** RIP assay using non-specific IgG or anti-Ago2 antibody. The relative enrichment of LINC00893 and miR-3173-5p was normalized to the input samples. **E** RNA pull-down assay using biotin-labeled miR-3173-5p probe or miR-NC control probe. The relative enrichment of LINC00893 was normalized to the input samples. **F** miR-3173-5p expression levels in 66 pairs of PCa tissues and para-cancerous tissues were assessed by RT-qPCR. **G** The correlation between LINC00893 and miR-3173-5p expression level was evaluated by Spearman correlation coefficient analysis. **H** The relative miR-3173-5p expression levels in PCa cell lines (PC-3, DU145, VCaP and LNCaP) and human normal prostate epithelial cell line RWPE-1 were detected by RT-qPCR. **I** miR-3173-5p expression levels were examined by RT-qPCR in cells transfected with pcDNA3.1-LINC00893 or vector. Three independent assays were performed with three technical replicates. The error bars are defined as s.d. *, P < 0.05, **, P < 0.01, and ***, P < 0.001

To investigate the subcellular localization of LINC00893, we performed nuclear and cytoplasmic fractionation and quantified the relative level of LINC00893 in these two cellular compartments. RT-qPCR analysis showed that LINC00893 was mainly localized in cytoplasm (Fig. 3A). To search for the downstream miRNA targets of LINC00893, we used Starbase online tool which predicted the binding site between LINC00893 and miR-3173-5p (Fig. 3B). To investigate their functional interaction, we conducted dual-luciferase reporter assay using reporter containing wild type binding site (WT) of miR-3173-5p and LINC00893 or reporter containing mutated binding site (MUT). The results demonstrated that miR-3173-5p mimic inhibited the luciferase activity of WT-LINC00893 reporter, while no effect was observed in MUT-LINC00893 reporter (Fig. 3C). To further confirm their physical interaction, we performed RNA Immunoprecipitation (RIP) using non-specific IgG or anti-Ago2 antibody (Ago2 is the indicator of lncRNA acting as a sponge and IgG serves as a negative control). RT-qPCR analysis showed that anti-Ago2 antibody enriched more LINC00893 and miR-3173-5p compared with IgG control (Fig. 3D). Further, RNA pull-down assay confirmed that Biotin-labeled miR-3173-5p probe enriched more LINC00893 than miR-NC control probe (Fig. 3E). Together, these results suggest the physical and functional interaction between LINC00893 and miR-3173-5p.

We next assessed miR-3173-5p expression levels in PCa tissues and para-cancerous tissues and found that miR-3173-5p level was upregulated in PCa tissues (Fig. 3F). Spearman correlation analysis further demonstrated a negative correlation between the expression levels of LINC00893 and miR-3173-5p (Fig. 3G). In addition, miR-3173-5p expression level was elevated in PCa cell lines (PC-3, DU145, VCaP and LNCaP) when compared to prostate epithelial cell line RWPE-1 (Fig. 3H). Importantly, LINC00893 overexpression significantly suppressed miR-3173-5p expression in both PC-3 and LNCaP cells (Fig. 3I). Taken together, the above results indicate that LINC00893 functions as a sponge to negatively regulate miR-3173-5p.

### MiR-3173-5p mediates the effect of LINC00893 overexpression in PCa

**Fig. 4:**
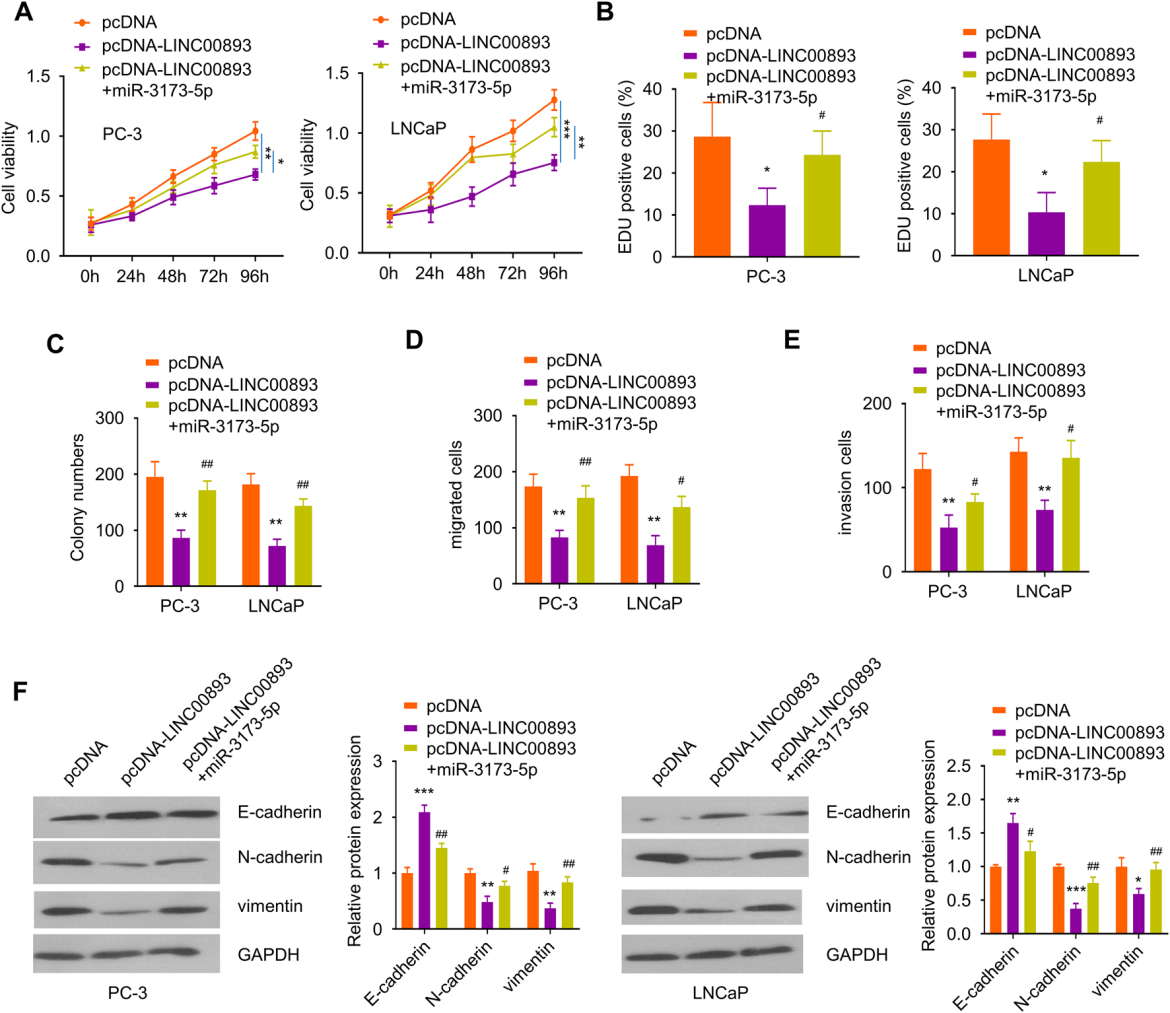
miR-3173-5p mediates the effects of LINC00893 on PCa cell phenotype. PC-3 and LNCaP cells were transfected with pcDNA vector, pcDNA-LINC00893 or pcDNA-LINC00893 + miR-3173-5p mimic for 48 h**. A**, **B** The proliferation ability of PCa cells under different conditions was detected by CCK-8 (**A**) and EdU incorporation assay (**B**). **C** Colony formation assay in PC-3 and LNCaP cells under the above experimental conditions. Transwell migration assay (**D**) and invasion assay (**E**) in PC-3 and LNCaP cells under the above experimental conditions. **F** EMT related proteins E-cadherin, N-cadherin and vimentin were evaluated by Western blot in PC-3 and LNCaP cells under the above experimental conditions. Three independent assays were performed with three technical replicates. The error bars are defined as s.d. *, P < 0.05, **, P < 0.01, and ***, P < 0.001

To demonstrate the functional engagement of miR-3173-5p in LINC00893 overexpression, PC-3 and LNCaP cells were transfected with pcDNA vector, pcDNA-LINC00893 or pcDNA-LINC00893 + miR-3173-5p-mimic. CCK-8 cell proliferation and EdU incorporation assay showed that miR-3173-5p overexpression by miR-3173-5p-mimic rescued cell proliferation upon LINC00893 overexpression (Fig. 4A and B). Consistently, miR-3173-5p overexpression also rescued the clonogenic ability upon LINC00893 overexpression (Fig. 4C), as well as cell migration ability (Fig. 4D) and cell invasion ability (Fig. 4E). miR-3173-5p overexpression also attenuated the increase of E-cadherin and increased the level of N-cadherin and vimentin in cells transfected with pcDNA-LINC00893 (Fig. 4F). Collectively, these data support the notion that LINC00893 regulates PCa progression through sponging miR-3173-5p.

### miR-3173-5p targets 3'UTR region of SOCS3 mRNA

**Fig. 5:**
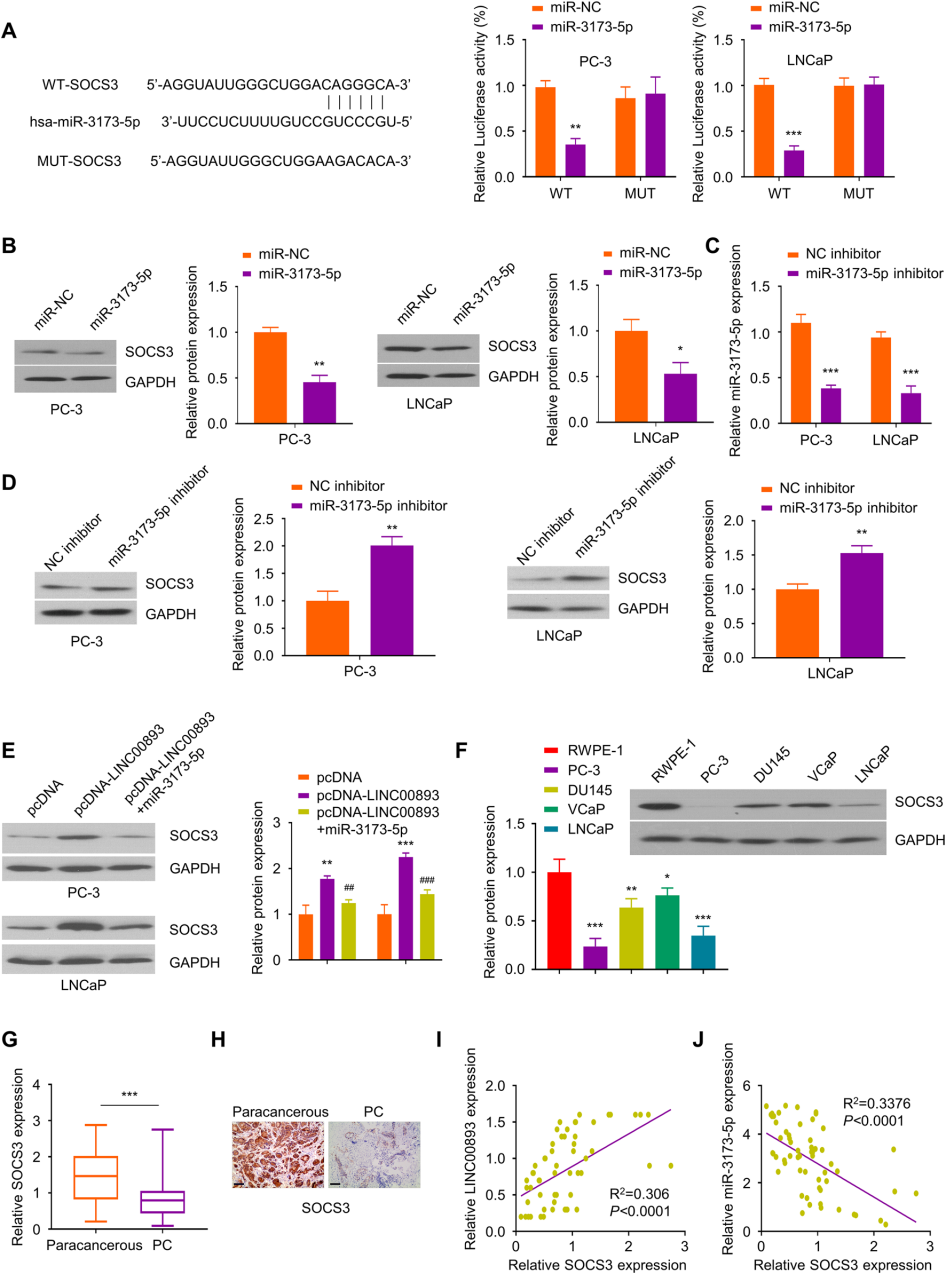
MiR-3173-5p binds to the 3'UTR of SOCS3 mRNA and inhibits its expression. **A** The binding sites of miR-3173-5p on SOCS3 mRNA 3'UTR region were predicted through Starbase database. Dual luciferase reporter assay was performed using pmirGLO-WT-SOCS3 and pmirGLO-MUT-SOCS3 reporter in the presence of miR-3173-5p mimic or miR-NC. Renilla luciferase control plasmids were co-transfected into cells, and the relative firefly luciferase activity in the reporter was normalized to renilla luciferase activity in the control plasmid. **B** SOCS3 protein levels were examined by Western blot after the transfection of miR-3173-5p mimic in PC-3 and LNCaP cells. **C** miR-3173-5p expression level was examined by RT-qPCR in PC-3 and LNCaP cells after the transfection with miR-3173-5p inhibitor or NC inhibitor. **D** SOCS3 protein levels were assessed by Western blot after the transfection with miR-3173-5p inhibitor or NC inhibitor. **E** Cells were transfected with pcDNA vector, pcDNA-LINC00893 plasmid or pcDNA-LINC00893 + miR-3173-5p mimic for 48 h, and SOCS3 protein levels were examined by Western blot. **F** SOCS3 protein levels in PCa cell lines and human normal prostate epithelial cell line RWPE-1 were evaluated by Western blot. **G** Relative SOCS3 mRNA levels in 66 pairs of PCa tissues and para-cancerous tissues were analyzed by RT-qPCR. **H.** SOCS3 protein levels in PCa tissues and matched para-cancerous tissues were evaluated by IHC. Scale bar: 100 μM. **I**,** J** The correlations between LINC00893 and SOCS3 mRNA, and between MiR-3173-5p and SOCS3 mRNA were analyzed by Spearman correlation coefficient analysis. Three independent assays were performed with three technical replicates. The error bars are defined as s.d. *, P < 0.05, **, P < 0.01, and ***, P < 0.001

To investigate the mRNA target of miR-3173-5p, we relied on starbase online tool to predict the binding sites between miR-3173-5p and the 3’UTR of SOCS3 mRNA. Dual luciferase reporter assay revealed that miR-3173-5p mimic inhibited the luciferase activity of pmirGLO-WT-SOCS3 reporter, but showed no effect on pmirGLO-MUT-SOCS3 reporter (Fig. 5A), which indicates the functional interaction between miR-3173-5p and SOCS3 mRNA. Consistently, the overexpression of miR-3173-5p reduced SOCS3 protein level (Fig. 5B). We also performed loss-of-function analysis using miR-3173-5p-inhibitor. The transfection of miR-3173-5p inhibitor significantly suppressed miR-3173-5p expression (Fig. 5C), but increased the expression level of SOCS3 protein (Fig. 5D). In addition, the overexpression of LINC00893 increased SOCS3 protein level, which could be suppressed by miR-3173-5p-mimic (Fig. 5E). SOCS3 expression levels were also found to be significantly lower in PCa cell lines and PCa tumor tissues (Fig. 5F–H). In addition, Spearman correlation coefficient analysis showed that there was a positive correlation between SOCS3 mRNA and LINC00893 level, and a negative correlation between SOCS3 mRNA and miR-3173-5p expression (Fig. 5I and J). Taken together, our data indicate that miR-3173-5p binds to 3'UTR of SOCS3 mRNA and suppresses its expression.

### Silencing SOCS3 partially alleviates the inhibitory effects of miR-3173-5p inhibitor on the malignant phenotype of PCa cells

**Fig. 6:**
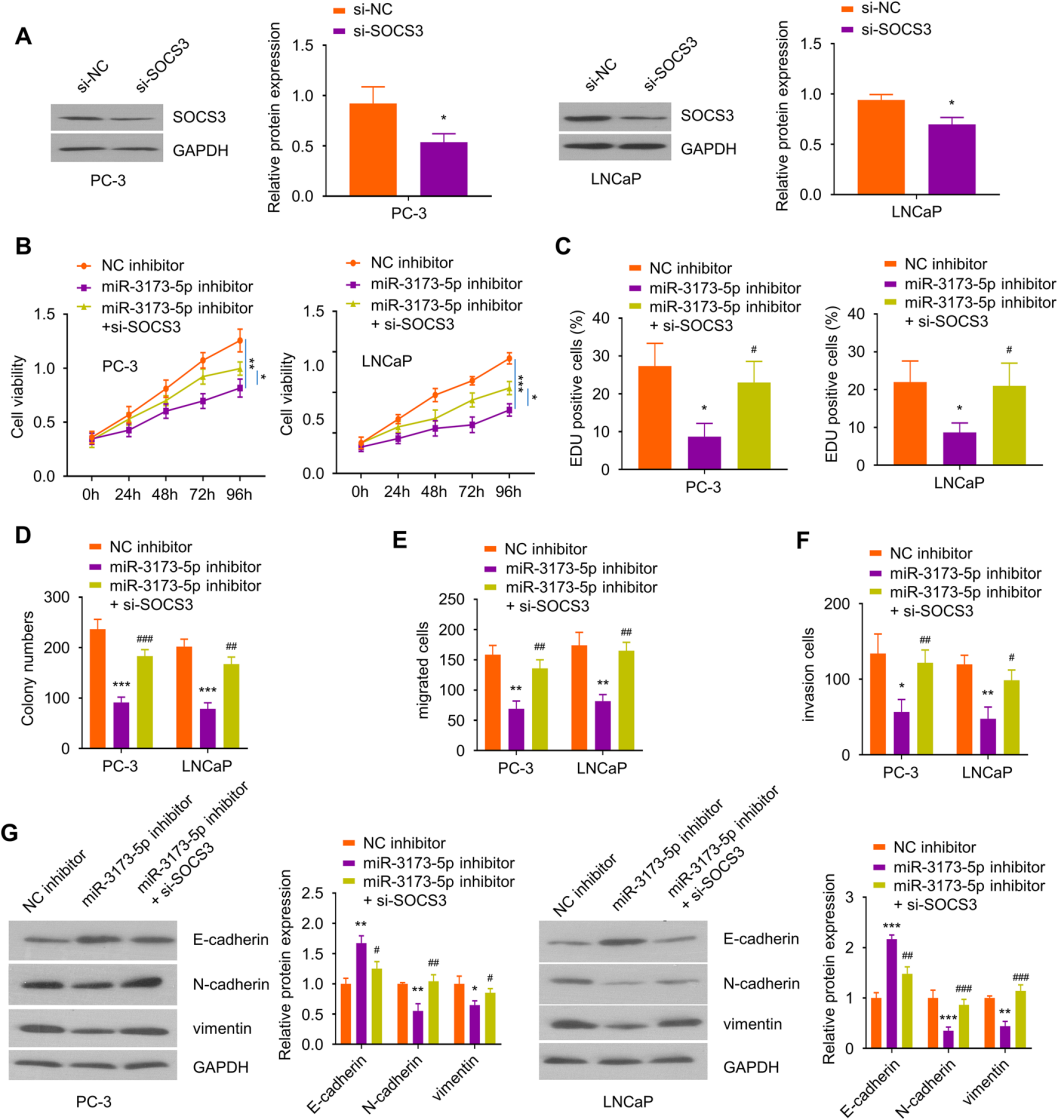
SOCS3 silencing attenuated the effects of miR-3173-5p inhibitor in PCa cells. **A** The knockdown efficiency of SOCS3 siRNA in PC-3 and LNCaP cells was examined by Western blot. For **B-G,** PC-3 and LNCaP cells were transfected with NC inhibitor, miR-3173-5p inhibitor or miR-3173-5p inhibtor + si-SOCS3. **B, C** The proliferation ability of cells in the above experimental groups was evaluated by CCK-8 (B) and EdU incorporation assay (**C**). **D** Colony formation assay in PC-3 and LNCaP cells of the above experimental groups. Transwell migration assay (**E**) and invasion assay (**F**) in PC-3 and LNCaP cells of the above experimental groups. **G** The protein levels of EMT markers E-cadherin, N-cadherin and vimentin in different treatment groups were detected by Western blot. Three independent assays were performed with three technical replicates. The error bars are defined as s.d. *, P < 0.05, **, P < 0.01, and ***, P < 0.001

To validate that SOCS3 is a downstream effector of miR-3173-5p, PC-3 and LNCaP cells were transfected with siRNA targeting SOCS3 (si-SOCS3) and matched controls (si-NC). Western blot showed that the transfection of si-SOCS3 effectively reduced SOCS3 level (Fig. 6A). We then transfected PC-3 and LNCaP cells with NC inhibitor, miR-3173-5p inhibitor or miR-3173-5p inhibitor + si-SOCS3. Functional assays demonstrated that miR-3173-5p inhibitor significantly impaired cell proliferation, clonogenic ability, cell migration and invasion ability, as well as the expression of EMT markers (Fig. 6B–G). However, silencing SOCS3 rescued these phenotypes as well as EMT marker expression (Fig. 6B–G). Collectively, the above results suggest that SOCS3 mediates the roles of miR-3173-5p in regulating the malignant phenotype of PCa cells.

### LINC00893 regulates SOCS3/JAK2/STAT3 axis through sponging miR-3173-5p.

**Fig. 7:**
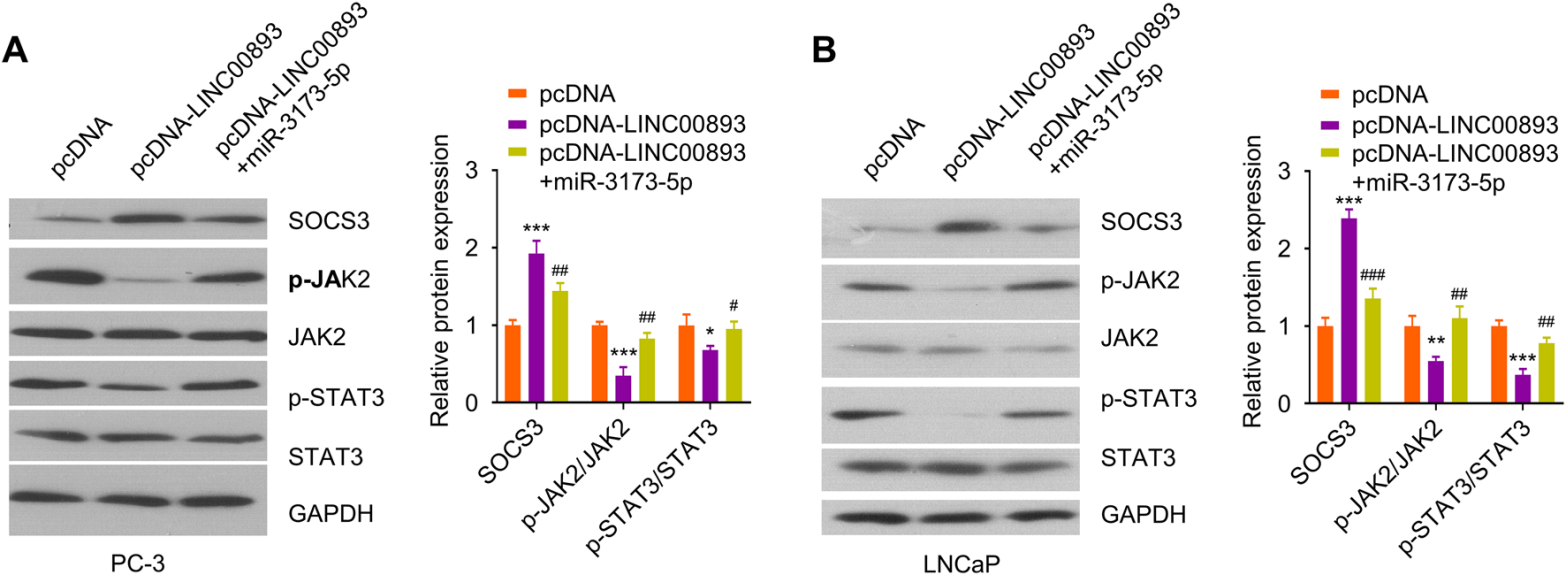
LINC00893/miR-3173-5p axis regulates SOCS3/JAK2/STAT3 signaling pathway. PC-3 and LNCaP cells were transfected with pcDNA, pcDNA-LINC00893 or pcDNA-LINC00893 + miR-3173-5p mimic. The protein levels of p-JAK2, p-STAT3, JAK2, STAT3 and SOCS3 in PC-3 (**A**) and LNCaP (**B**) cells were assessed by Western blot. Three independent assays were performed with three technical replicates. The error bars are defined as s.d. *, P < 0.05, **, P < 0.01, and ***, P < 0.001

To further explore the signaling pathway regulated by LINC00893, we transfected PC-3 and LNCaP cells with pcDNA3.1 vector, pcDNA3.1-LINC00893, or pcDNA3.1-LINC00893 + miR-3173-5p mimic. Since SOCS3 is a inhibitor for JAK2/STAT3 signaling pathway, we sought to examine the activation status of JAK2 and STAT3. Subsequently, we detected the protein levels of SOCS3, p-JAK2, JAK2, p-STAT3 and STAT3. The results showed that LINC00893 overexpression promoted SOCS3 level and inhibited the phosphorylation of JAK2 and STAT3. The co-transfection of miR-3173-5p mimic suppressed SOCS3 expression and enhanced the phosphorylation of JAK2 and STAT3 (Fig. 7A and B). Collectively, these data suggest that SOCS3/JAK2/STAT3 pathway is a target downstream of LINC00893 and miR-3173-5p.

### LINC00893 overexpression suppresses the tumorigenesis of PCa cells in vivo

**Fig. 8:**
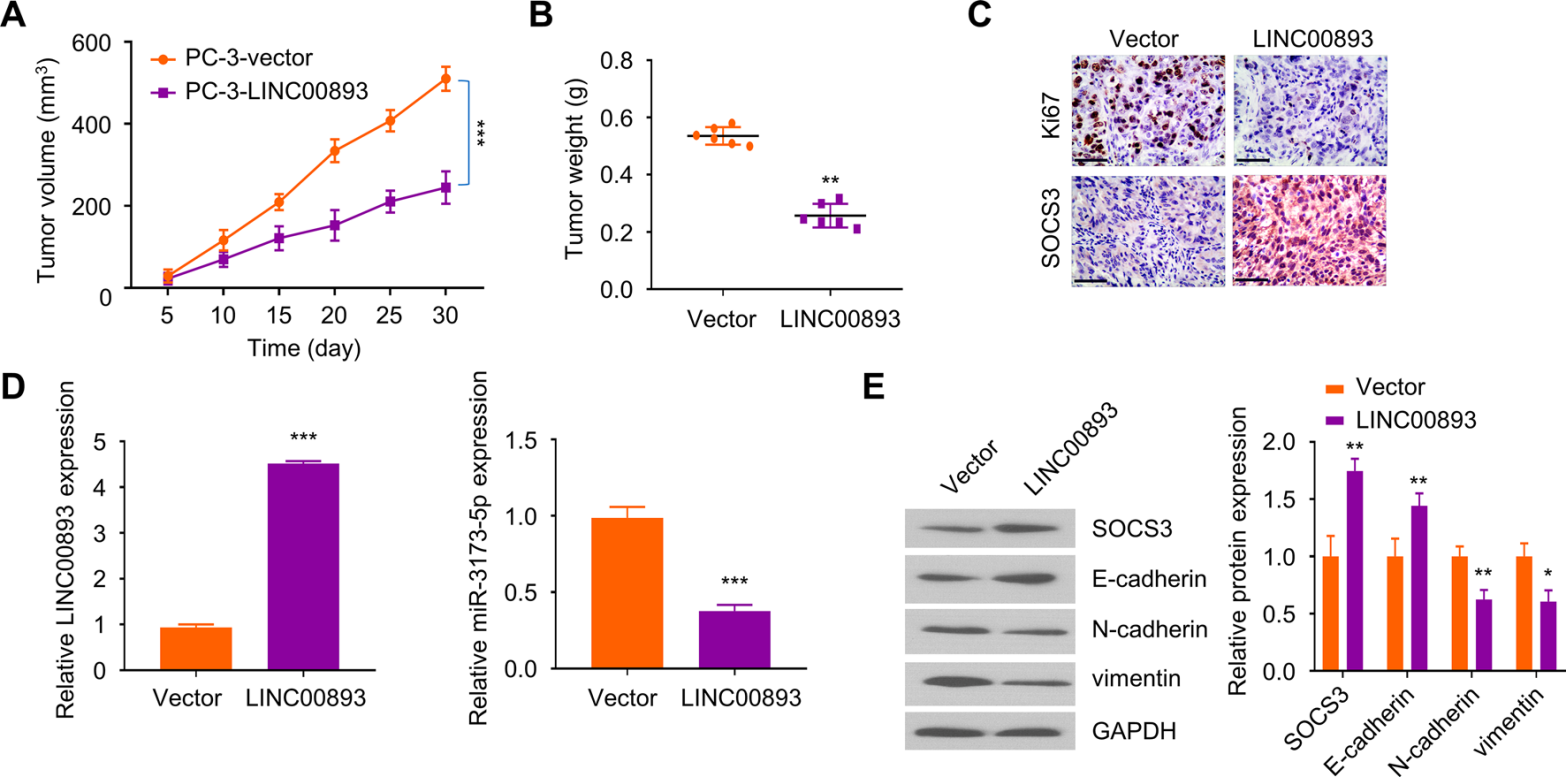
Overexpression of LINC00893 suppresses the tumorigenesis of PCa cells in nude mice. PC-3 cells stably expressing LINC00893 (PC-3-LINC00893) or the control cells (PC-3-vector) were inoculated subcutaneously into NOD/SCID mice (n = 6 in each group). **A** The volume of the xenograft tumor was measured every 5 days. **B** The weight of the subcutaneous xenograft tumor was measured on day 30. **C** LINC00893 and miR-3173-5p expression levels in xenograft tumor tissues were assessed by RT-qPCR. **D** The protein levels of SOCS3, E-cadherin, vimentin and N-cadherin in xenograft tumor tissues was detected by Western blot. **E** Ki-67 and SOCS3 protein expression levels in xenograft tumor sections were detected by IHC staining: Scale bar: 100 μM. Three independent assays were performed with three technical replicates. The error bars are defined as s.d. *, P < 0.05, **, P < 0.01, and ***, P < 0.001

Lastly, we conducted xenograft tumorigenesis assay in mice using PC3 cells stably expressing LINC00893 or PC3 cell with empty vector. The results showed that LINC00893 overexpression hindered the tumorigenesis of PC-3 cells in nude mice (Fig. 8A and B). We also extracted RNA from xenograft tumor samples, and RT-qPCR analysis revealed that the overexpression of LINC00893 significantly suppressed miR-3173-5p expression in tumor samples (Fig. 8C). Furthermore, the protein levels of SOCS3 and E-cadherin were upregulated, while N-cadherin and vimentin were downregulated in PC-3-LINC00893 tumor samples (Fig. 8D). In the meanwhile, we also performed IHC staining of cell proliferation marker Ki-67 and SOCS3 in tumor sections. SOCS3 staining level was much stronger while Ki-76 signal was diminished in xenograft tissues with LINC00893 overexpression (Fig. 8E). Taken together, The above results supported the notion that LINC00893 acts as a tumor suppressor to inhibit the tumorigenesis of PCa cells.

## Discussion

In summary, we found that LINC00893 was downregulated in PCa tissues and cell lines, and the low level expression of LINC00893 was closely associated with the TNM staging and distant metastasis of PCa. Patients with low LINC00893 expression level are also associated with a poor prognosis, which suggests a tumor suppressor role of LINC00893. Indeed, LINC00893 overexpression in PCa cells not only suppresses cell proliferation and the migratory capability in vitro, but also impairs the tumorigenesis of PCa cells in nude mice. Together, our data support the tumor-suppressor role of LINC00893 in regulating the progression of PCa.

We further demonstrated that LINC00893 sponges miR-3173-5p and negatively regulates its expression. The expression level of LINC00893 is negatively correlated with miR-3173-5p in PCa tissues, and miR-3173-5p mimic transfection could rescue the effects of LINC00893 overexpression. miR-3173-5p seems to bind to the 3'UTR region of SOCS3 mRNA, which suppresses SOCS3 expression. As a inhibitor of JAK2/STAT3 signaling pathway [30, 31], SOCS3 expression level negatively regulates the phosphorylation status of JAK2 and STAT3. Overall, our data unveils a novel tumor-suppressor role of LINC00893 in PCa by targeting miR-3173-5p and maintaining the expression of SOCS3.

Recent studies have implicated lncRNAs in the progression of PCa. For example, lncRNA AATBC enhances the proliferation and metastasis of PCa cellsby sponging miR-1245b-5p [22], and lncRNA CRNDE targets miRNA-146a-5p to regulate the progression of PCa [46]. LncRNA AFAP1-AS1 promotes PCa progression through the miR-15b/IGF1R (Insulin-like growth factor 1 receptor) axis [47]. A recent study showed that LINC00893 is downregulated in thyroid cancer (THCA) and inhibits the growth and metastasis of THCA cells by stabilizing PTEN and inhibiting the AKT pathway [48]. Our study adds novel evidence that LINC00893 functions as a tumor suppressor in PCa.

The dysregulation of miR-3173 is implicated in the progression of ovarian carcinoma [49] and B-cell acute lymphoblastic leukemia [50]. In B-cell acute lymphoblastic leukemia, the downregulation of miR-3173 promotes cell invasion and tumorigenesis. This is consistent with our observation that miR-3173-5p is downregulated in PCa and miR-3173-5p inhibitor promotes the cell proliferation of PCa cells. Interestingly, we found that miR-3173-5p acts as a downstream effector of LINC00893. However, the underlying mechanisms by which LINC00893 is downregulated in B-cell acute lymphoblastic leukemia and prostate cancers remian unclear, which warrants further investigation.

The aberrant activation of JAK2/STAT3 signaling pathway is associated with the progression of various tumors. In PCa, JAK2/STAT3 can regulate the malignant phenotype of cancer cells through rewiring metabolic pathways [51]. Furthermore, JAK2/STAT3 signaling pathway also serve as a crucial mediator for the anti-tumor effects of Scoparone [52], β-citric acid [53] and Atractylenolide II [54]. In lung adenocarcinoma, JAK2/STAT3 activation contributes to EMT and the metastasis of cancer cells [55]. In melanoma, the anti-tumor activity of brevilin A also depends on the status of JAK2/STAT3 pathway [56]. It has also been shown that JAK2 inhibitor can synergize with methylsulfonylmethane to curb the progression of bladder cancer [57]. As a crucial negative regulator of JAK2/STAT3 pathway, SOCS3 expression seems to be regulated by multiple mechanisms in PCa. For example, miR-2909 regulates the ISGylation system by inhibiting SOCS3 [58]. SOCS3 modulates the sensitivity of PCa cells to the targeted anticancer drug Enzalutamide [59]. miR-221 also regulates SOCS3 expression and is associated with the survival of high-risk PCa [60], and SOCS3 has been proposed as a prognostic factor in PCa patients [61]. SOCS3 overexpression in castration-resistant prostate cancer cells increases the sensitivity to the killing by natural killer cells [62]. Our data support the tumor-suppressor function of SOCS3 and highlights the role of SOCS3/JAK2/STAT3 axis in PCa progression.

Our study also hints several questions to be further explored. First, the mechanisms by which LINC00893 is downregulated in PCa cells remain to be elucidtaed. In addition, JAK2/STAT3 inhibitors should be applied in animal model to confirm their roles in regulating tumorigenesis upon LINC00893 overexpression. Furthermore, the genes or gene programs governed by JAK2/STAT3 signaling pathway in PCa cells need to be systematically investigated.

## Conclusions

In summary, this study clarified the role and mechanism of LINC00893 in regulating the progression of PCa. LINC00893 functions as a tumor suppressor to inhibit cell proliferation, tumorigenesis, migration and invasion of PCa cells. LINC00893 is downregulated in PCa tissues, which is associated with a poorer prognosis. LINC00893 binds to miR-3173-5p as a ceRNA, thereby impairing the inhibitory effect of miR-3173-5p on SOCS3 expression. The dysregulated SOCS3/ JAK2/STAT3 signaling pathway due to LINC00893 downregulation could contribute to the progression in PCa. Our research enriches the molecular network of LINC00893 in regulating the progression of PCa, and provides a theoretical foundation for PCa targeted therapy.

## Supplementary Information

**Additional file 1.** Western blot grayscale raw data.

## Data Availability

All data and materials were contained in the final version manuscript.
